# Evidence for Health III: Making evidence-informed decisions that integrate values and context

**DOI:** 10.1186/s12961-016-0085-4

**Published:** 2016-03-14

**Authors:** Anne Andermann, Tikki Pang, John N Newton, Adrian Davis, Ulysses Panisset

**Affiliations:** Department of Family Medicine and Department of Epidemiology, Biostatistics and Occupational Health, Faculty of Medicine, McGill University, Montreal, Canada; Lee Kuan Yew School of Public Policy, National University of Singapore, Singapore, Singapore; Institute of Population Health, Faculty of Medical and Human Sciences, University of Manchester, Manchester, England; Public Health England, London, England; Department of Preventive and Social Medicine-Health Policy, Faculty of Medicine, Federal University of Minas Gerais, Belo Horizonte, Brazil; Evidence Informed Policy Network (EVIPNet) Steering Group, World Health Organization, Geneva, Switzerland

**Keywords:** Decision-making, Evidence-based medicine, Health equity, Health outcomes, Health policy, Public health, Research

## Abstract

Making evidence-informed decisions with the aim of improving the health of individuals or populations can be facilitated by using a systematic approach. While a number of algorithms already exist, and while there is no single ‘right’ way of summarizing or ordering the various elements that should be involved in making such health-related decisions, an algorithm is presented here that lays out many of the key issues that should be considered, and which adds a special emphasis on balancing the values of individual patients and entire populations, as well as the importance of incorporating contextual considerations. Indeed many different types of evidence and value judgements are needed during the decision-making process to answer a wide range of questions, including (1) What is the priority health problem? (2) What causes this health problem? (3) What are the different strategies or interventions that can be used to address this health problem? (4) Which of these options, as compared to the status quo, has an added benefit that outweighs the harms? (5) Which options would be acceptable to the individuals or populations involved? (6) What are the costs and opportunity costs? (7) Would these options be feasible and sustainable in this specific context? (8) What are the ethical, legal and social implications of choosing one option over another? (9) What do different stakeholders stand to gain or lose from each option? and (10) Taking into account the multiple perspectives and considerations involved, which option is most likely to improve health while minimizing harms? This third and final article in the ‘Evidence for Health’ series will go through each of the steps in the algorithm in greater detail to promote more evidence-informed decisions that aim to improve health and reduce inequities.

## Background

**Fig. 1:**
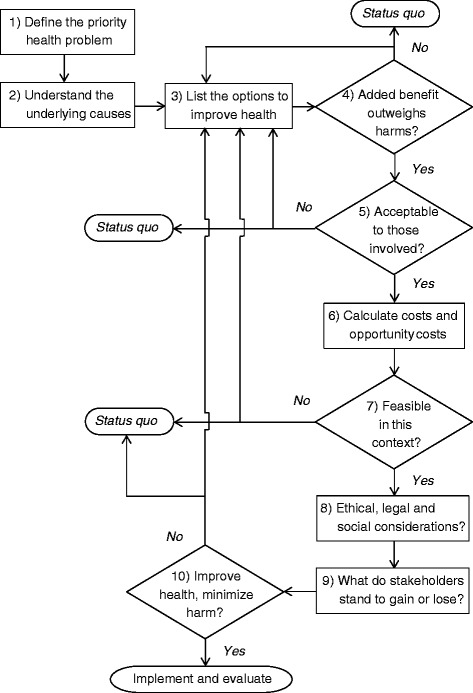
Algorithm for making evidence-informed decisions to improve health [1]

Making evidence-informed decisions with the aim of improving the health of individuals or populations can be facilitated by using a systematic approach [1]. Indeed, there is an entire field of scientific inquiry devoted to medical decision-making with a vast number of different types of algorithms and approaches which have been proposed for structuring the decision-making process [2-4]. While there is no single way of summarizing or ordering the various elements that should be involved in making such health-related decisions, the following algorithm proposed here lays out, in a straightforward and non-mathematical way, many of the key issues that should be considered, with a strong focus on evidence, values and context (Fig. 1). The remainder of this third and final article in the ‘Evidence for Health’ series will go through each of the steps in the algorithm in greater detail to promote more evidence-informed and nuanced decisions that aim to improve health and reduce inequities.

## Review

### Define the priority health problem

At the outset, it is helpful to try and identify the priority health problem (or problems) that, if improved, will have the greatest impact on health overall [5]. All too often, a new drug or other intervention is discovered and people lobby for its implementation and scale-up when the need for this intervention has not even been demonstrated and it is unclear whether it will truly have a widespread impact on the health of a large segment of the population. Therefore, it is important, in the first instance, that we understand what the true health needs are in order to be able to then address them in an evidence-informed way. An objective needs assessment based on surveillance data of the common causes of morbidity and premature mortality, combined with a subjective needs assessment based on what the community may consider the most important priorities, is a good way to start. For example, the Tanzania Essential Health Intervention Project involved a community-based research approach that identified the true causes of death in two districts in Tanzania (where most deaths occurred at home, thus rendering hospital data unreliable). The evidence was then used to adjust the allocation of the district health budget to be more in line with the real disease burden. Within 5 years, a 52% reduction in child mortality was noted and the two districts reached the related Millennium Development Goal targets well before 2015 [6].

### Understand the underlying causes

It is difficult to tackle a health problemunless there is an understanding of what causes the problem and where it would be possible to intervene to mitigate the problem or ideally to prevent the problem from occurring in the first place. For a long time, the focus has been on the proximal causes of health problems commonly known as the ‘risk factors’ (e.g. smoking, diet, exercise, etc.). More recently, Sir Michael Marmot coined the phrase “*the causes of the causes*” to show how the distribution of risk factors in a population can be influenced by other upstream factors known as the “*social determinants of health*” [7]. These include factors such as education, employment, family income, housing and social support, which influence the health status of populations and can help to explain why some people are healthy and others are not [8]. Going even further upstream, Jeff Reading, formerly Director of the Institute of Aboriginal People’s Health at the Canadian Institutes for Health Research described the “*causes of the causes of the causes*” to show how the Aboriginal determinants of health underlie the social determinants, which in turn underlie the distribution of risk factors, leading to the notion that there can be proximal, intermediate and distal causes of poor health [9]:

“*What’s the cause of diabetes? Many would say diet and exercise. The cause of that is poverty and lack of choice regarding diet and exercise. And the cause of that is colonization and lack of economic opportunity*” [10].

While increasing access to and quality of clinical services is clearly important, it cannot be the only approach to improving health. Rather, a broad range of strategies are needed, including engaging community leaders, end-users/consumers and other key stakeholders to create healthier physical and social environments, as well as incorporating culturally-adapted and contextualized solutions to local health problems: “*Diabetes is a complicated disease that is nested in the experience of rapid social and cultural change; thus, its prevention and control may need new ideas that go beyond an individual approach in a clinic or hospital ward*” [11]. Indeed, changing these distal determinants will require strong advocacy for larger social change and legal reforms, which will only occur if there is buy-in and support from the highest political levels.

### List the options to improve health

Once we have defined the health priorities and have understood the underlying causes of these problems at various levels, the next question to ask is “what can we do about it?” There is always more than one option available, including the status quo, but how to choose? The first step is simply identifying what the options are before attempting to weigh the pros and cons of each.

**Fig. 2:**
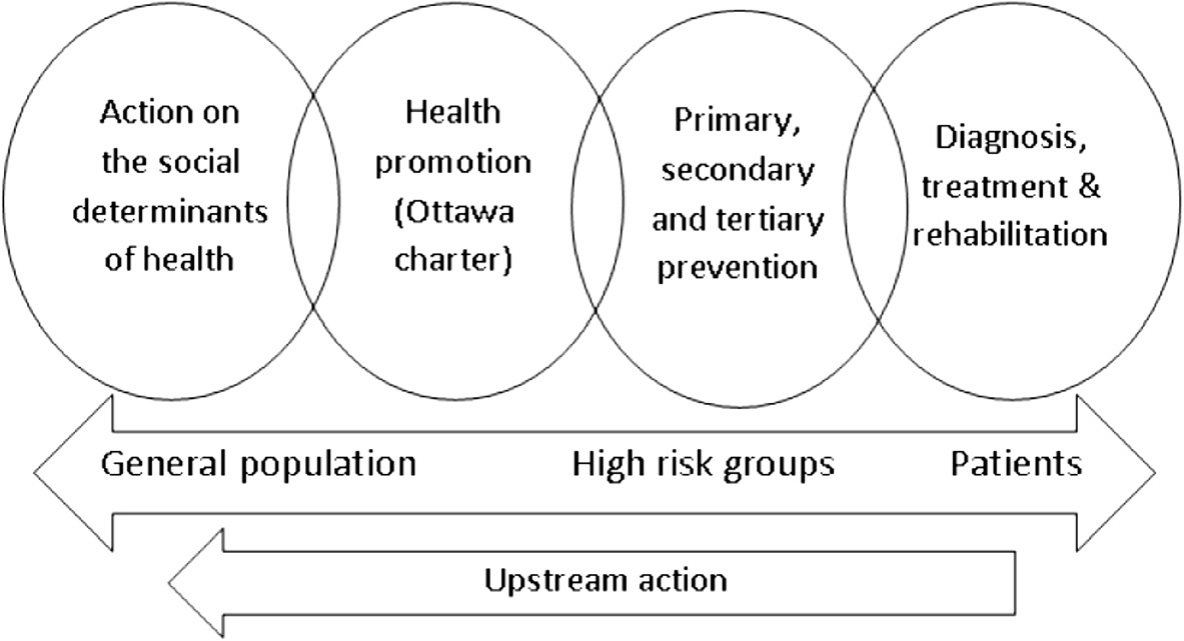
A continuum of strategies is required to improve health

At a population level, improving health requires a continuum of strategies (Fig. 2). All too often, when considering which strategies to choose, people stay within their comfort zone, screening for risk factors and counselling to promote behaviour change. Whether it is the prevention of obesity and heart disease or promoting smoking cessation, there is the notion that people should be making healthy choices and if they get sick then they are to blame. Yet, these strategies that focus on the more proximal causes fail to adequately address the intermediate and distal causes of poor health. Vineis and Elliot consider that being a “*prisoner to the proximate*” is “*a serious mistake*” [12]. Mackenbach agrees that we should not be blaming the individual since “*human health and disease are the embodiment of the successes and failures of society as a whole*” [13]. Moreover, there is a growing arsenal of strategies (such as intersectoral action, health in all policies and so forth) that focus on addressing the upstream determinants and changing the environment to make the healthy choices the easy choices. However, if these strategies are not included on the ‘list of options’ they will never be chosen. Indeed, working ‘upstream’ may actually bring even greater health improvements across a large number of areas by acting on the shared causes of poor health.

### Do the added benefits outweigh the harms?

To answer this question, it can be helpful to consider a number of sub-questions: What is the option being considered and what is it being compared to (i.e. the current gold standard or, if there is no gold standard, then the status quo)? Is there an added benefit at all? And, if so, what are the added benefits? Is there data on more distal benefits such as improvements in health outcomes or only intermediate and proximal measures, such as changes in patient or health provider behaviours, leaving one to extrapolate as to the actual impacts of such an intervention? Are there any harms? And, if so, what are the harms? Can one say with confidence that there are no or few harms, or was this something that was not measured, and therefore an area of uncertainty? How likely are the benefits and harms and for whom?

To answer these questions, syntheses of the existing evidence are often used, including systematic reviews and meta-analyses, which provide an overview of the existing evidence base. These studies are generally known as ‘secondary research’ since they do not involve primary data collection. Instead, the current body of evidence is used as the starting point, and the study involves asking a clearly formulated research question, systematically collecting research evidence around this question, critically appraising the research studies to determine which should be included, and finally integrating the results from a wide range of studies, to thereby present a clearer picture than any individual study alone can provide [14]. This often serves as an important anchor to decision-making, particularly in areas which are highly value-driven and open to debate. However, it can be difficult to assess the risk-benefit balance when the harms are not being measured. For instance, in the case of breast cancer screening, the literature on mortality benefit came first. As time went on and national breast screening programs were being implemented, only then did a new literature start to emerge on the potential harms of screening, ranging from psychological side effects and cancer worries [15] to the effects of radiation related to mammography itself [16]. As a result, this new evidence provided an opportunity for rethinking the balance of benefits and harms [17].

Indeed, determining whether there is an added benefit from the intervention which outweighs the harms often involves a value judgement and may vary according to one’s risk tolerance – how much a person wants to avoid getting the disease being targeted (e.g. breast cancer) and how much they want to avoid the potential negative consequences inherent to the intervention itself (e.g. false positive test result, unnecessary-treatment). Where there is widespread agreement in a given context that the added benefits outweigh the harms, we can continue on with our algorithm; however, if not, then it is back to the drawing board to look at other options from the list or to maintain the status quo while working on developing better options for the future.

### Are the options acceptable to those involved?

Before implementing any treatment, program or policy, it is important to think about those who will be ‘on the receiving end’. Since we cannot know people’s preferences, opinions or concerns unless we ask, it is therefore important that they be involved. Understanding individual and population preferences, as well as the local context of implementation, is critical to choosing the best option under the circumstances. No point thinking about costs if the option is not acceptable in the first place.

For example, some genetic screening programs entail providing information to be able to make more informed reproductive choices, and during pregnancy, that could involve the delicate issue of abortion. In some communities, due to religious beliefs or cultural values, having a screening program which includes the possibility of aborting an unborn child would be quite unacceptable, even if certain individuals within that community may want to have that avenue open to them if it turns out that their child is affected. Thus, the issue of acceptability can become quite tricky, especially when it involves balancing the preferences of individuals with those of the target population and society overall. Another example of where issues of acceptability are particularly sensitive is when the intervention is delivered to the entire population and there is no real possibility to ‘opt out’. For instance, fluoridation of water is widely recognized as a powerful means of improving oral health [18]. Yet, in spite of strong evidence in support of such programs [19], outcries from vocal opponents have resulted in millions of people not being protected against dental decay, and even the halting of certain community-based water fluoridation programs [20]. Thus, if none of the options are acceptable, then either further information can be provided, which may succeed in changing attitudes and generating support for these options, or else it may be a sign to go back to the drawing board and try to think up more options.

### Calculate costs and opportunity costs

Generally, we find ourselves in a situation where resources are scarce and, importantly, there is competition with priorities beyond the health sector. So, how do we determine which option to choose and which options need to be left behind (i.e. the opportunity cost)? The Disease Control Priorities Project [21] and the Choosing Wisely campaign [22] use effectiveness and cost-effectiveness data to determine which interventions to scale-up to improve global health and which interventions to scale back. The cost effectiveness ratios are generally much lower for preventive interventions as opposed to curative interventions. For instance, childhood immunization, insecticide-treated bed nets for malaria prevention and road traffic safety interventions (such as speeding laws and speed bumps at busy intersections) cost only a few dollars per DALY averted, whereas treating a stroke or heart attack with expensive ‘clot-busting’ medications can cost thousands of dollars per DALY averted.

Thus, action on the social determinants of health to prevent problems at the source (i.e. by improving housing, education, job quality, and so forth) are likely to be much less expensive as compared to treating people once they are ill, with all the healthcare costs, missed work and human suffering that this entails. That being said, it is often difficult to make such calculations for interventions that address the social determinants of health as these tend to involve more complex ‘clusters’ of interventions with less clarity in terms of the specific costs and outcomes [23]. Indeed, the evidence base relating to the costs of working more upstream are often lacking, even if evidence of effectiveness is growing. That does not mean that we should not be working more upstream, simply that it is not always easy to estimate the costs involved. Moreover, when engaging in intersectoral action and whole-of-government approaches, it also remains to be determined which budgetary envelope these costs should be withdrawn from (i.e. the health budget or the education budget, the national budget or the local budget, and so forth). Often, within government, ministries of health tend to have less bargaining power, especially in low- and middle-income countries, which poses a challenge when it comes to making health improvement a priority. Further, there can be “*inter-governmental downloading of responsibilities*” between national, district and municipal levels [24]. Nonetheless, while cost issues are clearly important, they are not the only factor in determining the feasibility of one option over another.

### Which options are feasible in this context?

Even if an option is affordable and can be funded using existing budgets, this does not necessarily mean that it is feasible since there may be many different kinds of logistical barriers involved, which can often be difficult to assess in advance. Indeed, just because an intervention works well in one context or setting does not mean that it will be applicable and transferable to a different one. There are many factors that can influence whether it is likely to expect the same benefits in this new implementation context [25]; for instance, if the health issue is just as prevalent and important as in the original study population, if there is strong local capacity for implementing the intervention, if the intervention can be suitably adapted to the new social and cultural context, and so forth.

Across many parts of Africa and South East Asia, while it may be possible to afford the cost of vaccines against childhood diseases – especially as much of the cost is subsidized by the GAVI alliance through the Expanded Programme on Immunization – there are nonetheless various logistical challenges that may remain. Vaccination attempts may be thwarted by social or political unrest, by difficult access to remote areas, and by lapses in the cold chain [26]. Even if a program is successfully launched, ongoing sustainability remains a concern once initial funding is depleted, governments change and attention turns to other priorities. Piloting would therefore be needed, sometimes also termed a ‘feasibility study’, to first provide evidence of local effectiveness and potential for long-term health gains.

### Ethical, legal and social considerations

In some cases, such as end-of-life issues or genetic screening, the ethical, legal and social issues are quite apparent [27]. However, it may be even more important to proceed with caution in situations where such issues are less obvious and could potentially be overlooked. For instance, an economic development project that proposes to create more jobs in a rural area may seem harmless enough. Yet, what if only a small minority benefit from such an initiative, or even worse, what if those who benefit least are burdened with most of the harms? Kass, who developed an ethics framework for public health, emphasized the importance of ensuring that interventions not only lead to greater benefit than harm, but also ensuring that the distribution of benefits and harms is equitable [28].

Thus, while there may be high quality evidence of efficacy and low cost, this in itself is not sufficient to move ahead with one option over another. It is important to carefully consider the ethical, legal and social implications and to systematically examine who is affected and in what way, to ensure that everyone is able to share in the benefits while especially protecting those who are most vulnerable from the harms. This brings us to our penultimate step on understanding what the various stakeholders stand to gain or lose.

### What do stakeholders stand to gain or lose?

When considering the potential impacts of any given option, it is important to look at the different groups who could be affected – including various lobby groups, target populations, innocent bystanders and the decision-makers themselves – to better appreciate what each group would gain or lose from implementing this option and what would be the distribution of the benefits and harms.

Many policies outside the health sector (e.g. tax laws, parental leave policies, employee disability insurance schemes, etc.) can have tremendous potential to greatly benefit and/or harm the health of various groups in society. This is the impetus behind the “*Health in All Policies*” approach championed by Ilona Kickbush et al. [29], and also the growing interest in conducting Health Impact Assessments to help create more evidence-informed policy [30].

Returning once again to the example of an economic development project being proposed in a remote community, it would certainly be unfair if the company shareholders retained the bulk of the profits while the workers exposed to potentially hazardous conditions are paid low wages. Similarly, if community leaders do not redistribute the wealth and other benefits from deals signed with the company or at least use the funds to benefit the local population in some way, that would also be unfair. Indeed, lack of transparency and profit sharing, lack of equal opportunities for employment, lack of inclusion and empowerment of those affected, as well as failure to reinvest profits into health and social services and tackling the upstream determinants of health (e.g. through improving education, access to child care, building social cohesion, etc.) could all lead to worsening health inequities – in spite of an influx of money and jobs in the region. Knowing all this, prior to the last step in the algorithm, there is an opportunity for refining or even redesigning the proposed option such that the benefits are maximized, the harms are minimized, and the distribution of benefits and harms is more equitable. Thus, once these additional safeguards and adjustments are incorporated, only then is it possible to determine which options have the greatest potential for improving health and reducing inequities.

### Which options improve health most while minimizing harms?

At the end of the day, a decision must be made that integrates all of the evidence from the various steps in the algorithm and makes a final judgement regarding whether or not to proceed. The ultimate question is “which option(s) maximize potential health and social benefits, minimize potential harms, and ensure that the distribution of benefits and harms within and between populations is fair?” Many different types of evidence and expertise are called upon to make this overarching decision. Clearly, there will be differences in how one goes about this depending on whether these are clinical decisions, population level decisions or global policy decisions.

Integrating the evidence, values and contextual factors to come to a final decision is indeed a difficult challenge; even with all the information at hand, the challenge is how to take the plunge and transform all of these different considerations into a binary choice – “will I choose this option or that option?” There are examples, especially in clinical settings, of decision-aids with explicit value clarification exercises which have been shown to lead to more informed value-based choices [31] and to decrease decisional conflict [32]. However, especially in policy settings, information cannot simply be plugged into a formula or equation to come up with the best answer. Indeed, there needs to be a structure that involves the various stakeholders in a deliberative process and, by following the algorithm outlined in this chapter, they will be better able to have a more logical and structured discussion that teases out the complexity involved and avoids recourse to polemics and entrenched views, which are often counter-productive. Often, the process itself is part of the product – by helping to ensure that different voices are heard, that people feel respected, and that a fair and transparent process is used to come to a decision [33], it will be possible to make more reflected judgements about improving health and reducing inequities based on the best available evidence and taking into account a multitude of complex considerations.

## Conclusions

As Michael Marmot once said, “*Scientific findings do not fall on blank minds that get made up as a result. Science engages with busy minds that have strong views about how things are and ought to be*” [34]. While no decision is ever free from value judgements, at least using a systematic approach ensures that the decision is nonetheless grounded in the best available evidence, in all its facets. Additionally, by identifying and making explicit the various trade-offs and opportunity costs, and by providing reasons for choosing one option rather than another, at least there will be the possibility of understanding why certain value judgements were made, as well as being able to challenge the underlying reasons in future should the context or evidence-base evolve.

It is important to strike a balance between making decisions that are not at all thought through versus overthinking every step. The systematic approach can therefore be helpful to avoid becoming too bogged-down in the details to see the big picture, or even worse, being paralyzed by indecision when faced with a large number of perspectives and trade-offs. In the end, decisions must be made, since failing to choose is simply choosing the status quo. Being transparent and explicit about the basis for making the decision, including the quality of evidence, is an important requirement. There are also the very helpful support tools developed by Lavis et al. [35] which aim to help in bridging the ‘know-do’ gap.

The goal is therefore to foster a dialogue among stakeholders that will promote decisions that are more nuanced, more transparent and, ultimately, more likely to have an impact on improving health. Nonetheless, decision-making remains an inherently iterative and often somewhat disorganized process, especially as we move towards population-based and global-level decisions. According to Anderson, a policy analyst, “*Public policy is messy. If you hold on too tightly to your policy formulation you will wither in this environment. Policy is rarely final and usually changes with every new administration*” [36].

Therefore, all the more reason to ensure that decisions are informed by evidence and that the key stakeholders are involved in a meaningful way.
